# Psychiatry Curricula 2022

**DOI:** 10.1192/bjo.2021.449

**Published:** 2021-06-18

**Authors:** Pauline Whitelaw, John Russell

**Affiliations:** Royal College of Psychiatrists

## Abstract

**Aims:**

The aim of our review was to ensure that:

Curricula are aligned to the GMC's GPC and Excellence by Design Frameworks

Curricula are capability focused

Curricula promote a flexible and adaptable approach to training

Curricula are succinct, user friendly, patient-centred and reflective of current training in practice

**Background:**

In response to recommendations outlined in the Shape of Training Review (2013), the GMC developed their new framework for postgraduate medical education Excellence by Design (2015), alongside their Generic Professional Capabilities (GPC) Framework (2015).

**Method:**

Governance

To manage the review, a Curriculum Revision Working Group (CRWG) was set up to monitor and govern the review process. Members include Specialty Advisory Committee (SAC) chairs, trainee and patient/lay representatives.

Curriculum Development & Framework

The CRWG, alongside SACs and specialty working groups, have undertaken a “Why, What, How” approach in developing the curriculum framework. Each curriculum is structured as follows:

High Level Outcomes (HLOs) – These outline the “Why”, and provide an overarching view on what should be achieved by trainees. Each HLO is mapped directly onto each of the nine GMC GPC domains.

Key Capabilities – These outline the “What”, and provide key detail on what trainees need to undertake to fulfil specific aspects of the curriculum.

Training illustrations – These outline the “How”, and supplement the Key Capabilities by providing real-world examples of how to achieve each capability.

Development of the curricula included:

Mapping current Intended Learning Outcomes (ILOs) to the new HLO framework

Re-writing competencies so that they were capability focused

Undertaking a thematic analysis of the curricula, to develop key themes/groupings for capabilities

Review and update Workplace Based Assessments (WBPAs) to ensure they align to the new framework

Stakeholder Engagement

Part of the review has been to ensure Key Stakeholders are involved at each stage of curriculum development. To ensure that all key stakeholders are provided opportunity for consultation, a stakeholder map was developed.

Stakeholder engagement has included:

Direct trainee/trainer/patient/lay involvement at curriculum review meetings

Consultation surveys at each development stage, including feedback on the draft curriculum framework and feedback on full draft curricula

Attendance at meetings with key stakeholders, including NHS Employers and Royal College meetings

**Result:**

The review is currently ongoing. In 2020 we were successful in submitting all 10 of our curricula to the GMC for approval. We are continuing to further develop our curriculum framework, which includes:

- Psychiatry “Silver Guide”
- Curricula documents
- Training illustrations
- ARCP Decision Aids
- Supplementary Guidance

**Conclusion:**

The review of RCPsych curricula has provided an excellent opportunity to broaden curriculum capabilities, and ensure that the curricula are achievable and deliverable. Our aim is to ensure that the new curricula promote flexibility and adaptability within training, and are user friendly for both trainees and trainers.

